# Phenolic extract from olive mill wastewater sustains mitochondrial bioenergetics upon oxidative insult

**DOI:** 10.1016/j.fochms.2024.100234

**Published:** 2024-12-11

**Authors:** Iolanda Rita Infantino, Salvatore Antonio Maria Cubisino, Stefano Conti Nibali, Paola Foti, Marianna Flora Tomasello, Silvia Boninelli, Giuseppe Battiato, Andrea Magrì, Angela Messina, Flora Valeria Romeo, Cinzia Caggia, Vito De Pinto, Simona Reina

**Affiliations:** a Dept. of Biomedical and Biotechnological Sciences, University of Catania; b We.Mitobiotech S.R.L; c Council for Agricultural Research and Economics (CREA)– Research Centre for Olive, Fruit and Citrus Crops, Acireale, CT, Italy; d Dept. of Agriculture, Food and Environment (Di3A), University of Catania, Catania, Italy; e Institute of Crystallography, National Council of Research, Catania Unit, Catania 95126, Italy

**Keywords:** Oil waste recovery, Phenolic compounds, Mitochondrial biogenesis, Oxidative stress, Mitochondrial respiration

## Abstract

In the last few years, many efforts have been devoted to the recovery and valorization of olive oil by-products because of their potentially high biological value. The olive mill wastewater (OMWW), a dark-green brown colored liquid that mainly consists of *olive* fruit vegetation water, is particularly exploited in this regard for its great content in phenolic compounds with strong antioxidant properties. In our previous work, we produced different OMWW fractions enriched in hydroxytyrosol- and hydroxytyrosol/oleuropein (i.e. C and OPE extracts, respectively) that exhibited considerable anti-microbial and radical-scavenging activities in vitro. Based on these findings, the present study aimed to assess the impact of C and OPE samples on mitochondrial function and oxidative stress response in mouse fibroblast-like cells (NCTC). Accordingly, OMWW phenolic extracts proved to enhance mitochondrial biogenesis and to reduce cellular sensitivity to hydrogen peroxide. Moreover, high-resolution respirometry experiments first time revealed the efficiency of OMWW phenols recovered by selective resin extraction in preventing mitochondrial respiration failure upon oxidative insult. Collected data definitely demonstrate the bioactivity of our phenolic-rich fractions, supporting the advantages of reusing the olive mill wastewater to generate, at low-cost, high added value molecules that could be useful for the improvement of health and nutrition products.

## Highlights

• Phenol-enriched OMWW extracts protect cells from ROS and promote mitobiogenesis.
• Oroboros O2k analysis reveal they ameliorate oxygen consumption upon H_2_O_2_ insult.
• OMWW fractions aim to distinct target market according to their processing method.

## 1 Introduction

Secoiridoid oleuropein (OLE) and its degradation derivative hydroxytyrosol (HT) are widely considered to be the major bioactive phenolic compounds responsible for the numerous health benefits of extra virgin olive oil (EVOO) (Hayes, Allen, Brunton, O'Grady, & Kerry, 2011), due to their antioxidant and anti-inflammatory properties (Impellizzeri et al., 2011; Kucukgul, Isgor, Duzguner, Atabay, & Kucukgul, 2020; Micheli et al., 2023; Mnafgui et al., 2015). Consistently, OLE has been reported to decrease oxidative and nitrosative stress (De la Puerta, Martínez Domínguez, Ruíz-Gutíerrez, Flavill, & Hoult, 2001) by promoting the expression of antioxidant enzymes via the activation of Nuclear factor erythroid 2-related factor 2 (NrF2) transcription (Martínez-Huélamo, Rodríguez-Morató, & Boronat, 2017; Sun et al., 2017) and by increasing the level of non-enzymatic antioxidants, such as ascorbic acid, glutathione, β-carotene and α-tocoferol (Al-Azzawie & Alhamdani, 2006; Hassen, Casabianca, & Hosni, 2015). NrF2, in turn, stimulated the mitochondrial biogenesis pathway through the expression of mitochondrial DNA (mtDNA), peroxisome proliferator-activated receptor gamma coactivator 1a (PGC-1α) and complexes II and IV of the electron transport chain (Dinkova-Kostova & Abramov, 2015; Gureev, Shaforostova, & Popov, 2019; Hayashi et al., 2017). Similarly, it has been demonstrated that HT avoided oxidative damage (Posadino et al., 2021) by simultaneously modulating mitochondrial biogenesis and detoxifying enzyme (i.e. superoxide dismutase) activity (Zhu et al., 2010). Notwithstanding, OLE and HT are considered double-edged swords as they inhibited cell proliferation and promoted apoptosis in several tumor cell lines via diverse mechanisms (Costantini, Di Sano, & Barbieri, 2020; Luo et al., 2013; Scicchitano et al., 2023; Sirianni et al., 2010; Yao et al., 2014). Barbieri and co-workers have shown that concentrations of hydroxytyrosol higher than 250 μM led to an increase in reactive oxygen species (ROS) that prevented metastatic progression and triggered cell death in melanoma cells (Costantini et al., 2020). The group of Faniello have confirmed that high doses of oleuropein exhibited cytotoxic and pro-apoptotic function in an in vitro model of ovarian and breast cancer cells, although at small doses it diminished ROS levels in one of the cell lines examined (Scicchitano et al., 2023).

Although hydroxytyrosol and oleuropein from olive oil are well absorbed in humans, dietary intake and bioavailability are low due to their substantial waste during the olive fruit extraction processes. The weak hydrophobicity of these compounds drives their accumulation in olive mill wastewaters (OMWW), one of the largest residues of the olive sector which have lately received a great deal of attention as inexpensive and valuable source of ingredients to be included in functional foods or to formulate new supplements with high added value (Cuffaro et al., 2023; Foti et al., 2022; Martínez-Zamora, Peñalver, Ros, & Nieto, 2021). When managed as waste, the huge amount of OMWW that is annually produced during the milling process of olives from November to February (over 30 million m^3^), represents a serious issue for Mediterranean countries. Hence, extensive studies have been carried out to find an effective strategy to valorize it (Dermeche, Nadour, Larroche, Moulti-Mati, & Michaud, 2013; Enaime, Dababat, Wichern, & Lübken, 2024; Foti et al., 2021), converting “waste” into “raw material”. The extraction of phenols from OMWW provides, on one side, environmental benefits due to the promotion of sustainable management of by-products that would otherwise represent dangerous pollutants, on the other, an economic source of high value-added compounds and revenue improvement for the agri-food companies (Faustino et al., 2019; Gómez-García, Campos, Aguilar, Madureira, & Pintado, 2021). These compounds, in fact, have wide-ranging application in food, pharmaceutical and cosmetic industries. Specifically, in the food industry, phenols have been suggested as functional preservatives able to increase the shelf life and the nutraceutical value of different products (meat, dairy, fish, bakery products and juices) (Foti et al., 2021; Foti et al., 2022). In search of new strategies to optimize the recovery of natural bioactive components to be used as nutraceutical ingredients, we have already characterized two hydroxytyrosol- and hydroxytyrosol/oleuropein-rich extracts obtained from OMWW by reverse osmosis (C sample) or selective resin extraction (OPE sample) (Foti et al., 2023). Both samples proved to be active against various pathogenic bacterial strains and to antagonize the deleterious outcomes of H_2_O_2_-induced oxidative stress on the electrophysiological features of human mitochondria porin 1 (VDAC1) embedded in artificial lipid membranes. These encouraging results prompted further investigations in continuous cell cultures.

In the present study, we focused on the effects of OMWW phenolic samples on mitochondrial bioenergetics exploiting mouse fibroblast-like cells (NCTC) challenged with hydrogen peroxide. To this aim, we monitored the efficacy of C and OPE extracts in reducing oxidative stress-induced cell death and in modulating mitochondrial biogenesis using the Incucyte Live-Cell Analysis System. In addition, accurate measurements of mitochondrial respiration were performed with the Oroboros Oxygraph-2 K (O2K) high-resolution respirometer to evaluate the strength of HT- and HT/OLE-rich samples in sustaining oxygen consumption following H_2_O_2_ exposure and preserving mitochondrial respiration from oxidative damage.

## 2 Materials and methods

### 2.1 Chemical characterization of phenolic extracts

C and OPE samples were subjected to chemical characterization as previously described (Foti et al., 2023). In particular, pH and total soluble solids (TSS) were determined. The pH value was measured trough a pH meter (Mettler DL25, Mettler Toledo International Inc., Columbus, OH, USA), while the TSS value, expressed as °Brix, was measured with a refractometer (Atago, RX-5000 Thermo Fischer Scientific, Milan, Italy). The content of total phenols (TP) was obtained through the Folin–Ciocalteu's colorimetric method. The absorbance of the mixed solution was spectrophotometrically measured at 765 nm (Cary 100 Scan UV–Visible, Agilent, Santa Clara, CA, USA) and results were expressed as mg gallic acid equivalents (GAE)/L of sample. In addition, phenols and organic acids were detected trough HPLC system in specific conditions (Foti et al., 2023). The used apparatus was a Waters Alliance 2695 HPLC liquid chromatography with Waters 996 photodiode array (PDA) detector, set at 280 nm and with Waters Empower software (Waters Corporation, Milford, MA, USA). At least *n* = 3 independent experiments (biological replicates) were conducted with *n* = 3 technical replicates.

### 2.2 Cell culture and treatments

The ATCC CCL-1 fibroblast cell line NCTC Clone 929 (L cell, L-929, derivative of Strain L) derived from *Mus musculus* connective tissues, was cultured in Dulbecco's Modified Eagle Medium (DMEM) (Gibco – Thermo Fisher Scientific Corporation, Waltham, MA, USA) supplemented with 10 % (*v*/v) fetal bovine serum (FBS) (Gibco – Thermo Fisher Scientific Corporation, Waltham, MA, USA) and 1 % (v/v) penicillin-streptomycin (Pen-Strep) (Sigma Aldrich, St. Louis, MO, USA) at 37 °C and 5 % CO_2_. Cell line was used at early passages (8–15 population doublings). Treatment with phenolic extracts was performed as previously described (Foti et al., 2023): cells were incubated with C 1:1000 (v/v) or OPE 1:500 (v/v) for 24 h. 50 μM Hydroxytyrosol (Sigma Aldrich, St. Louis, MO, USA) was used as control. To investigate the protective effects of OMWW extracts against oxidative stress, pre-treated cells were exposed to 1 mM H_2_O_2_ (Sigma Aldrich, St. Louis, MO, USA) for additional 1 h in serum-free medium. At least *n* = 3 independent experiments (biological replicates) were conducted with *n* = 5 technical replicates.

### 2.3 Quantitative real-time PCR

To evaluate mitochondrial biogenesis, mRNA expression levels of PGC1-α, TFAM and NRF-1 were measured by RT-qPCR (Mastercycler® ep realplex Real-time PCR System - Eppendorf, Amburg, Germany). Total RNA was extracted from approximately 1*10^6^ cells according to manufacturer's instructions using the TRIzol® reagent (Thermo Fisher Scientific, Waltham, Massachusetts, USA) and quantified on a UV–Vis spectrophotometer at λ = 260 nm. 1 μg of each RNA sample was subjected to reverse transcription using the QuantiTect Reverse Transcription Kit (Qiagen, Hilden, Germany) and amplified by RT-qPCR with the QuantiTect SYBR Green PCR Kit (Qiagen, Hilden, Germany). Primer pairs used for qPCR amplification are listed in Table 1**.** Thermocycling program was the same described in (Reina et al., 2022). Relative *gene expression* levels between treated and untreated samples were calculated with the 2^-ΔΔCT^ method (Livak & Schmittgen, 2001) using β-actin as reference gene for normalization. At least n = 3 independent experiments (biological replicates) were conducted with *n* = 4 technical replicates.

**Table 1:** List of primer pairs used for RT-qPCR.

**Fw PGC1- α**	5’-GATGCGCTGACAGATGGAGA-3’
**Rev PGC1- α**	5’-TAGCTGAGTGTTGGCTGGTG-3’
**Fw TFAM**	5’-CGCAGGAAAAGCTGAAGACT-3’
**Rev TFAM**	5’-TGTGCGACGTAGAAGATCCT-3’
**Fw NRF-1**	5’-AGTGGCAGCTTCTCAGGAC-3’
**Rev NRF-1**	5’-ACTCCAGTAAGTGCTCCGAC-3’
**Fw β-actin**	5’-ACACTGTGCCCATCTACGAG-3’
**Rev β-actin**	5′- AATGTCACGCATTTCCC-3’

### 2.4 Western blot analysis

NCTC cells were harvested at 70 % confluence from T-25 flasks and lysed with 50 mM Tris, 150 mM NaCl, 1 mM EDTA, 1 % Triton-X100 and 1 % protease inhibitor cocktail (Thermo Fisher Scientific, Waltham, MA, USA), pH 7.4. Equal amounts of each protein samples (40 μg) were loaded onto a 12 % SDS- polyacrylamide gel, electrophoresed and transferred to an *Amersham*™ *Hybond*® *P 0.45* μm PVDF membrane *(*GE Healthcare Life Sciences, Chicago, Illinois, USA). The blot was blocked in 1× TBS, 0.1 % Tween-20 containing 5 % *w*/*v* nonfat-dried milk bovine (Sigma Aldrich, St. Louis, MO, USA) for 1 h at RT and subsequently incubated overnight at 4 °C with the following primary antibodies: anti-SDHA (Abcam ab14715, 1:1000), anti-β-actin (m Abcam 8226, 1:1000). IRDye® 800CW Goat anti-Mouse IgG (LICOR 92632210, 1:10000) was used as secondary antibody. Protein bands were detected using the iBright™ CL 1500 Imaging System. The intensity of each protein band was quantified using Image Studio Lite software (Li-COR Biosciences, Lincoln, NE, U.S.A.). Measurements were repeated 3 times on gels from 3 independent experiments (biological replicates).

### 2.5 High resolution respirometry

The oxygen consumption rates in NCTC cells upon C, OPE and HT treatments and/or in the presence of H_2_O_2_ were analyzed by high-resolution respirometry (HRR) technique by the Oroboros O2k-FluoRespirometer (Oroboros Instruments, Innsbruck, Austria). A Substrate-Uncoupler-Inhibitor Titration (SUIT) protocol, developed for intact cells, was applied to measure the endogenous respiration (ROUTINE), the uncoupled respiration (LEAK) by the addition of the ATP synthetase inhibitor oligomycin (0.5 μM), the maximal capacity of the electron transport (ET) system (maximal ET capacity) upon titration with the protonophore carbonyl cyanide 3-chlorophenylhydrazone (CCCP, 0.5 μM), and the residual oxygen consumption (ROX) achieved with sodium azide (100 mM). All substrates were purchased by Sigma Aldrich (St. Louis, MO, USA). All the experiments were performed in MiR05 (Oroboros Instruments, Innsbruck, Austria) at 37 °C under constant stirring. From *n* = 4 to *n* = 5 independent experiments (biological replicates) were conducted with *n* = 3 technical replicates.

### 2.6 Respirometric data analysis

Instrumental and chemical background fluxes were calibrated as a function of the oxygen concentration using DatLab software (v7.4.0.1, Oroboros Instruments, Innsbruck, Austria). The oxygen consumption rates, corresponding to ROUTINE (R), LEAK (L), and maximal ET capacity (E) were corrected for the ROX and expressed as pmol/s per million cells or as a function of the maximal ET capacity (Flux Control Ratios, FCRs), as described in Doerrier et al. (2018).The ATP-related flux was calculated as R-L, while the R-Reserve was calculated as (R-L)/E, according to (Risiglione et al., 2020).

### 2.7 Live Cell Imaging & Analysis

**Cell proliferation** was monitored in real-time using the IncuCyte® Sx1 Live-Cell imaging platform (Sartorius, Göttingen, Germany) located within a 37 °C, 5 % CO_2_ cell incubator Essen BioScience Sartorius Incucyte S3 (Sartorius, Göttingen, Germany). Fibroblasts were seeded at a density of 70 × 10^3^ cells/well in a 48-well plate. OMWW extracts were administered 24 h post-seeding (6 replicates were performed for each treatment), and immediately imaged. Photomicrographs were taken every 3 h over a 48-h period. Data analysis focused on the initial 24 h due to reaching a growth plateau thereafter. Quantitative assessment of cell growth was performed using the IncuCyte® Live-Cell Imaging & Analysis Software (Sartorius, Göttingen, Germany), utilizing the basic analyzer module and representing results as *a percentage of cell confluence.*

To evaluate **radical oxygen species (ROS) production**, CellROX™ Green Reagent (Thermo Fisher Scientific, Waltham, MA, USA) was used as per manufacturer instructions. Green fluorescence was measured in 6 replicates of cultured fibroblasts, initially seeded at 70 × 10^3^ cells/well in a 48-well plate and grown in presence of HT, C or OPE for 24 h. Subsequently, cells were loaded with 2.5 μM CellROX™ Green and exposed or unexposed to 1 mM of hydrogen peroxide for 1 h. Photomicrographs were captured using the IncuCyte® Live-Cell Imaging & Analysis Software, with CellROX™ fluorescence quantified at 490/516 nm following background subtraction via the surface fit method of the basic analyzer module. Results were expressed as *Green Integrated Intensity per image (micron square)/phase area confluence per image (micron square).*

**Mitochondrial mass** was assessed using MitoTracker™ Green FM (MGT) (Thermo Fisher Scientific, Waltham, MA, USA) following manufacturer guidelines. Briefly, 200 nM MitoTracker™ Green FM was added to the wells and incubated (37 °C/5 % CO_2_/humidified air) for 30 min. Following incubation, MTG fluorescence (490/516 nm) was measured in 6 replicates of cultured fibroblasts seeded at 70 × 10^3^ cells/well in a 48-well plate and grown in presence of HT, C or OPE for 24 h. Photomicrographs were captured using the IncuCyte® Live-Cell Imaging & Analysis Software, with MTG fluorescence quantified following background subtraction using the surface fit method of the basic analyzer module. Results were expressed as *Green area per image (micron square)/phase area confluence per image (micron square).*

At least *n* = 3 independent experiments (biological replicates) were conducted with *n* = 5 technical replicates.

### 2.8 Statistical analyses

Data were statistically validated using GraphPad Prism (9.0.0) software and presented as function of the mean value (± SD) for each considered condition. Statistical significance was determined using Ordinary one-way ANOVA with Dunnett's multiple comparison test or *T*-Test for each experiment. Significance was obtained after evaluation of *P* values less than 0.05 (*), 0.01 (**), 0.001 (****) and 0.0001 (****).

## 3 Results

### 3.1 Phenolic extracts are non-toxic to NCTC cells

**Fig. 1:**
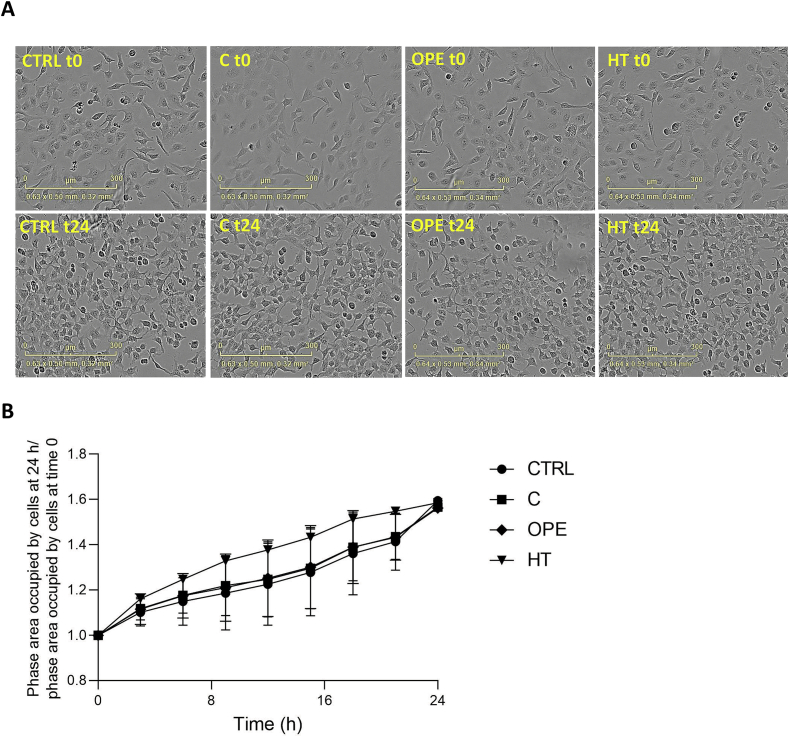
**A.** Phase contrast images of NCTC cells treated with 1:1000 *v*/v C, 1:500 v/v OPE or 50 μM HT taken at time 0 and after 24 h treatment. **B.** Growth curves of NCTC cells treated with 1:1000 v/v C, 1:500 v/v OPE or 50 μM HT and monitored in real time by using the IncuCyte® SX1 Live-Cell imaging system over 48 h. Cell growth curves during the first 24 h, calculated using the basic analyzer module of the Incucyte software, were reported as a percentage of cell confluence. Data are expressed as mean ± SD of three independent biological experiments, each with five technical replicates (*n* = 5) and compared to not-treated cells (Ctrl).

**Supplementary Fig. S1:**
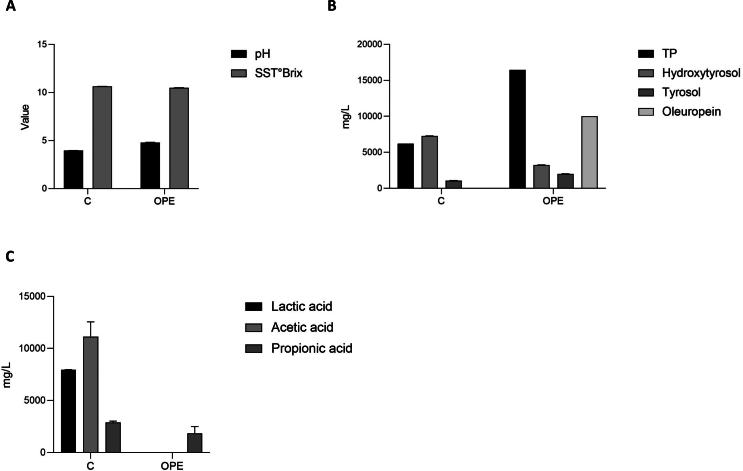
Chemical characterization of C and OPE samples. **A.** Determination of pH values and total soluble solids (TSS, expressed as °Brix) of the OMWW extracts. **B.** Measurement of total phenols (TP, expressed as mg gallic acid equivalents (GAE)/L of sample) and quantification of HT, T and OLE. **C.** Evaluation of lactic acid, acetic acid and propionic acid within samples. Data are indicated as mean ± SD of three independent biological experiments, each with three technical replicates (n = 3).

Considering the antiproliferative and pro-apoptotic effects of HT and OLE reported elsewhere (Costantini et al., 2020; Luo et al., 2013; Scicchitano et al., 2023; Sirianni et al., 2010; Yao et al., 2014), before deepening into the exploitability of C and/or OPE samples as nutraceuticals, we first monitored their potential toxicity on NCTC cell growth and morphology. The choice of this particular cell type, derived from mouse connective tissue, was essentially dictated by its large application in cytotoxicity assays (Hosseini et al., 2018; Rihani et al., 2018). Each OMWW extract, whose chemical composition is detailed in Suppl. Fig. 1, was diluted so that the hydroxytyrosol concentration supplied to the cells was 50 μM, as previously described (Gallardo-Fernández et al., 2019; Tagliafierro, Officioso, Sorbo, Basile, & Manna, 2015). Accordingly, cells were cultured in presence of 1:1000 (*v*/v) C, 1:500 (v/v) OPE or 50 μM HT alone and imaged in real time by using the Incucyte® system. Results depicted in Fig. 1B demonstrate that, within 24 h, both tested samples do not influence the overall rate of cellular biosynthesis neither produce detectable cell morphological changes (Fig. 1A). HT, used as control, slightly increases cell growth already after 2–4 h from the beginning of the treatment, without affecting cell shape. Outcomes of phenol compounds were monitored up to 48 h (not shown) proving the complete lack of harmful effects in the cell model selected.

### 3.2 Phenolic extracts protects NCTC cells from oxidative stress

**Fig. 2:**
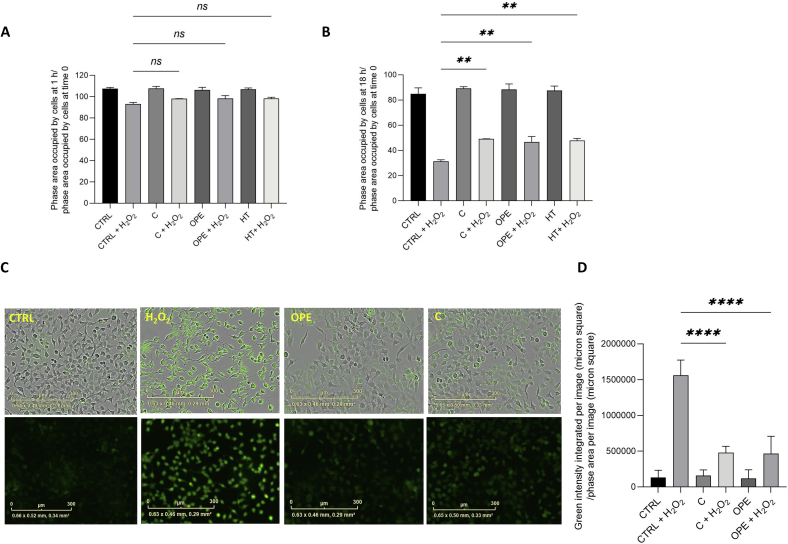
**A-B**: Cell proliferation/toxicity assay of NCTC cells pre-treated or not (Ctrl) with 1:1000 v/v C, 1:500 v/v OPE, or 50 μM HT for 24 h before being exposed to 1 mM H2O2 for 1 h (A) or 18 h (B). Results were expressed as the ratio of the phase area occupied by cells at 1 h or 18 h to the phase area occupied by cells at 0 h. **C.** Measurement of total ROS production via staining with CellROX™ Green Reagent. After 24 h treatment with the compound of interest, NCTC cells were loaded with 2.5 μM CellROX™ Green and subsequently exposed or not to 1 mM H_2_O_2_ for 1 h. The probe fluorescence was quantified at 490/516 nm following background subtraction via the surface fit method of the basic analyzer module. Results were expressed as Green Integrated Intensity per image (micron square)/phase area confluence per image (micron square). Data are expressed as mean ± SD of three independent biological experiments, each with five technical replicates (n = 5) and compared to not-treated cells (Ctrl); ** *p* < 0.01 and **** *p* < 0.0001. (For interpretation of the references to colour in this figure legend, the reader is referred to the web version of this article.)

The powerful antioxidant properties of olive phenols are claimed in countless reports (Bucciantini, Leri, Nardiello, Casamenti, & Stefani, 2021). In particular, both HT and OLE have proven capable of directly scavenging free radicals (Marković, Torić, Barbarić, & Brala, 2019; Rosignoli, Fuccelli, Sepporta, & Fabiani, 2016; Serreli & Deiana, 2020; Zorić, Kopjar, Rodriguez, Tomić, & Kosalec, 2021) following pro-oxidant stimuli in a concentration-dependent manner (Bigagli et al., 2017; Ilavarasi, Kiruthiga, Pandian, & Devi, 2011; Kitsati, Mantzaris, & Galaris, 2016; Li et al., 2022). To evaluate the efficacy of OMWW extracts in fighting oxidative damage and managing ROS overproduction under stressful conditions, NCTC cells were pre-incubated for 24 h in medium containing C, OPE or hydroxytyrosol alone at the concentrations selected based on earlier assessment of their cytotoxicity. Thence, the consequences of exogenous hydrogen peroxide administration were evaluated by using the Incucyte® Live Cell Analysis system. Fig. 2 displays the amount of cell survival upon 1 mM H_2_O_2_ exposure for 1 h (A) and 18 h (B). As can be observed in Fig. 2 A, one hour tratment with hydrogen peroxide results in a small drop (approximately 5 %) in cell viability, which, however, corresponds to a huge rise (roughly 7 times greater than control) in the total ROS detected by CellROX Green Reagent (Fig. 2C,D). Both phenolic samples greatly counteract the H_2_O_2_-induced imbalance in reactive oxygen species dramatically restoring values close to the untreated ones, although their protective role is not appreciable on cell survival most likely because of the scarce effect of hydrogen peroxide in the short time. On the contrary, C and OPE, similarly to HT, strongly reduce cell vulnerability to the pro-oxidant agent by incrementing cell survival of approximately 20 % upon long-term oxidative stress (Fig. 2B).

### 3.3 C and OPE stimulate mitochondrial biogenesis

**Fig. 3:**
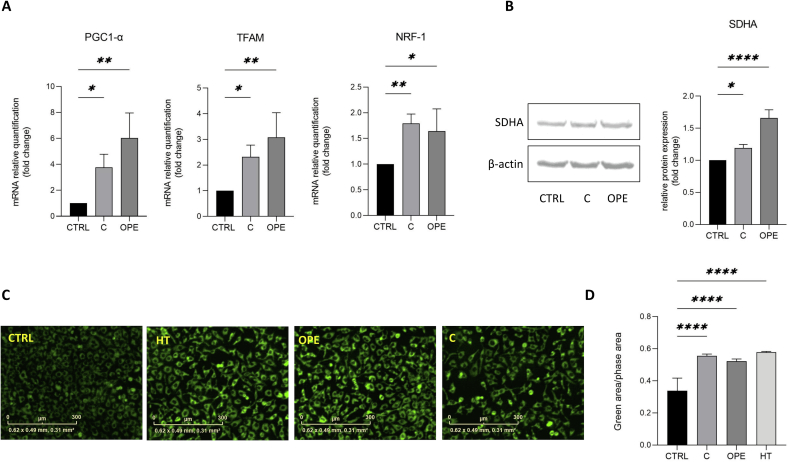
**A.** Relative quantification of PGC-1α, NRF1 and TFAM (mitochondrial biogenesis markers) mRNAs in NCTC cells treated with 1000 *v*/v C or 1:500 v/v OPE samples. Data are normalized to the β-actin, expressed as mean ± SD of three independent biological experiments, each with four technical replicates (*n* = 4) and compared to not-treated cells (Ctrl). **B.** Quantification of the mitochondrial content. Western blot illustration and relative quantification of mitochondrion-specific protein SDHA level in NTCT cells treated with 1000 v/v C or 1:500 v/v OPE extracts. Data are normalized to the β-actin, expressed as means ± SD of three independent biological experiments (*n* = 3) and compared to not-treated cells (Ctrl). **C.** Measurement of mitochondrial content by using the MitoTracker® Green MGT probe. Fluorescence was measured in NCTC cells treated with1:1000 v/v C, 1:500 v/v OPE or 50 μM HT for 24 h by using the IncuCyte® Sx1 Live-Cell imaging platform. Data are expressed as mean ± SD of three independent biological experiments, each with five technical replicates (n = 5) and compared to not-treated cells (Ctrl); * *p* < 0.05, ** *p* < 0.01 and **** *p* < 0.0001. (For interpretation of the references to colour in this figure legend, the reader is referred to the web version of this article.)

Cumulative evidence describes polyphenols as the primary natural compounds that stimulate mitochondrial biogenesis (Chodari et al., 2021; Hao et al., 2010). Thus, we investigated the effects of C and OPE samples on mitochondrial content by evaluating the PGC-1α (peroxisome proliferator-activated receptor gamma coactivator 1a) signaling cascade. PGC-1α governs mitochondrial respiration and biogenesis through the regulation of several transcription factors including NRF-1(nuclear respiratory factor-1) that, in turn, coactivates the mitochondrial transcription factor A (TFAM) (Gleyzer, Vercauteren, & Scarpulla, 2005; Kelly & Scarpulla, 2004; Viña et al., 2009). As shown in Fig. 3A, cell exposure to C and OPE extracts significatively enhances the mRNA levels of PGC1-α, TFAM and NRF-1 genes. This finding is supported by western blot analysis of the mitochondrial mass marker Succinate Dehydrogenase Complex Flavoprotein Subunit A (SDHA) (Olkhova et al., 2023) that confirms C and even more OPE sample positively modulate mitochondrial mass (Fig. 3B). Quantitative analysis of mitochondrial content by using the Incucyte® Live Cell Analysis System demonstrates once again the effectiveness of OMWW extracts in promoting growth and division of mitochondria: the MitoTracker™ Green FM Dye fluorescence is almost doubled in both C and OPE-exposed cell cultures, likewise to what obtained with HT alone (Fig. 3D).

### 3.4 HT and OPE partially reverts the impaired respiration of NCTC cell exposed to H_2_O_2_

**Fig. 4:**
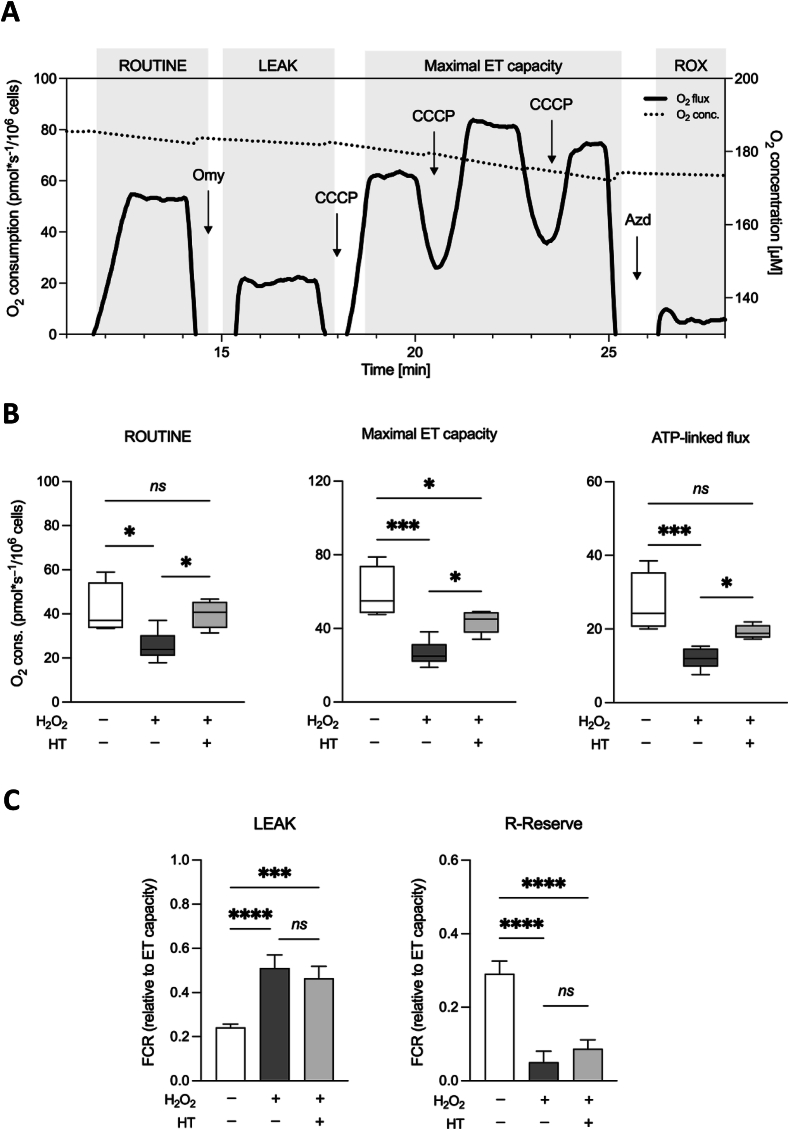
**A.** Representative curve displaying the respirometry profile of untreated NCTC cells and the SUIT protocol applied. The respiratory states Routine, Leak, Maximal ET capacity and ROX were achieved in intact cells with the specific addition of substrates and inhibitors, as following: Omy, oligomycin; CCCP, carbonyl cyanide 3-chlorophenylhydrazone; AZ, sodium azide. **B—C.** Quantitative analysis of the oxygen consumption in the analyzed states of NCTC cells pre-treated or not with 50 μM HT for 24 h and subsequently exposed to 1 mM H_2_O_2_ for 1 h. Data are indicated as mean ± SD Data are expressed as mean ± SD of five independent biological experiments, each with three technical replicates (*n* = 3); * *p* < 0.05, *** *p* < 0.001 and **** *p* < 0.0001.

**Fig. 5:**
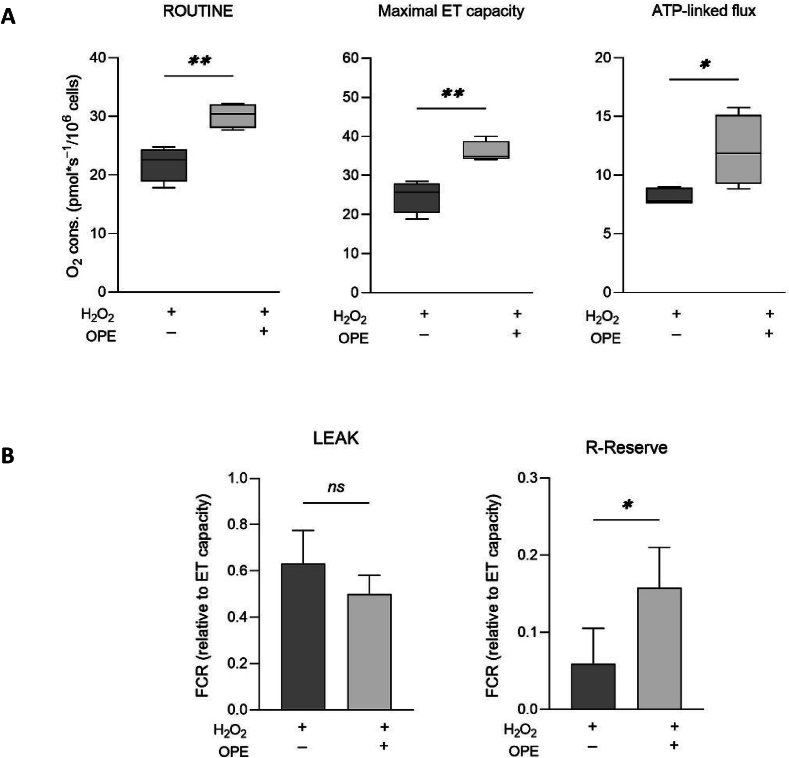
**A-B.** Quantitative analysis of the oxygen consumption in the analyzed states of NCTC cells pre-treated for 24 h with 1:500 *v*/v OPE and then exposed to 1 mM H_2_O_2_ for 1 h. Data are indicated as mean ± SD of five independent biological experiments, each with three technical replicates (n = 3); * *p* < 0.05 and ** *p* < 0.01.

**Supplementary Fig. S2:**
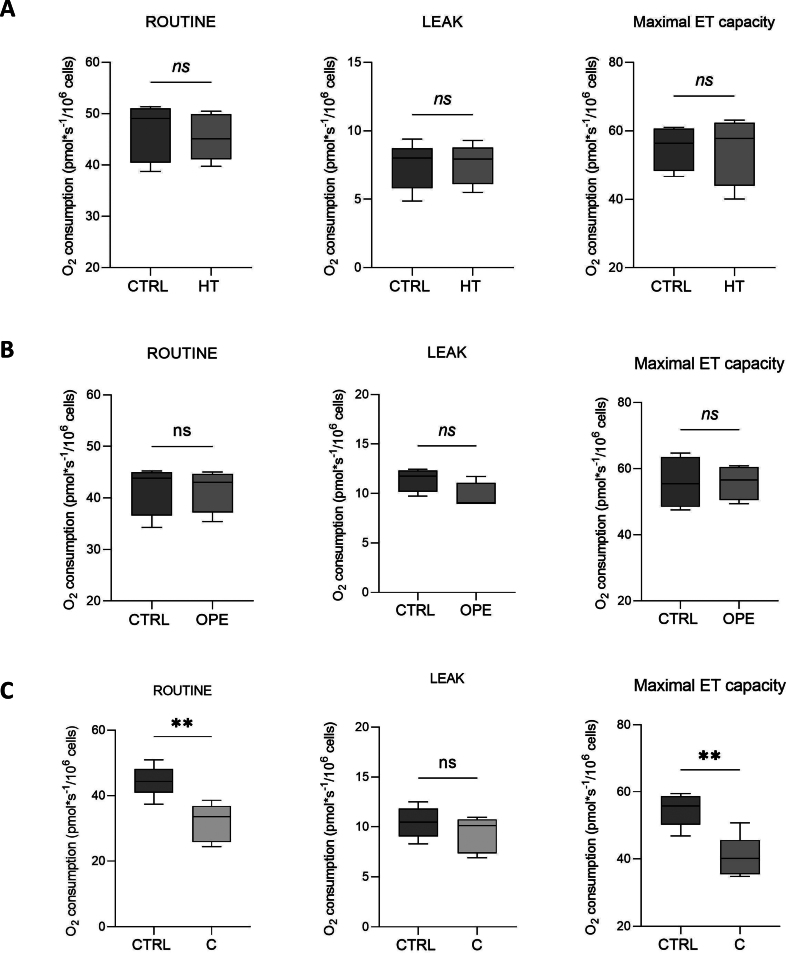
Quantitative analysis of the oxygen consumption in the analyzed states of NCTC cells treated with 50 μM HT (A), 1:500 v/v OPE (B) or 1:1000 v/v C (C) for 24 h. Data are indicated as mean ± SD of four independent biological experiments, each with three technical replicates (n = 3); ** *p* < 0.01.

Despite the numerous data regarding the promotion of mitochondrial biogenesis and functionality, detailed information about the effects of polyphenols on the activity of mitochondrial respiratory chain are missing. To fullfill this aspect, we analyzed the ability of our compounds to counteract H_2_O_2_ toxicity by using HRR. Fig. 4A displays a representative oxygen consumption curve relative to the untreated cells along with the SUIT protocol here applied. As shown in Fig. 4B, H_2_O_2_ dramatically affects the overall respiratory profile of NCTC cells, reducing of about 40 % and 55 % the ROUTINE and maximal ET capacity, respectively, as well as the respiration directly linked to ADP phosphorylation. In addition, as reported in Fig. 4C, the exposure to H_2_O_2_ doubles the LEAK contribution to the maximal ET capacity, correspondent to the dyscoupled respiration, while reduces of about 6 times the R-reserve. This term refers to the difference between basal respiration (oxygen consumption rate before addition of oligomycin) and maximal respiration (oxygen consumption rate after addition of CCCP) and represents the mitochondrial capacity to deal with a sudden increase in energy demand (for example due to an acute cellular stress) by producing extra ATP. R-Reserve is therefore a marker of mitochondrial fitness closely linked to bioenergetic health. Then, HRR experiments were repeated in presence of HT. While the HT treatment per se does not perturb the mitochondrial respiration (Suppl. Fig. 2A), upon H_2_O_2_ exposure it is able to partially restore the oxygen consumption rates corresponding to all the respiratory states here analyzed and the ATP-linked flux (Fig. 4B), but not the LEAK and R-Reserve FCRs (Fig. 4C). By using the same experimental approach, we next investigated the potential use of C and OPE for the scope. Firstly, we assayed their effect on NCTC cells, by measuring the respiration after 24 h of treatment. As shown in Suppl. Fig. 2B-C, while OPE does not show any significant impact on the mitochondrial respiration, in a similar manner to HT, C negatively affects the respiration, as it reduces both ROUTINE and the maximal ET capacity. Based on these results, the C sample was judged unsuitable for further analysis. OPE extract was indeed tested for its ability to counteract H_2_O_2_-induced mitochondrial injury. Similarly to what we observed for HT, OPE is able to increase the overall respiratory profile of NCTC cells exposed to H_2_O_2_ (Fig. 5A) as well as the R-Reserve (Fig. 5B). Here, the NCTC cells treated with hydrogen peroxide are assumed as control in order to highlight the recover in mitochondrial respiratory parameters induced by OPE treatment. Altogheter, these results indicate the partial ability of HT and OPE to avoid the harmful effect of H_2_O_2_ on mitochondrial respiration.

## 4 Discussion and conclusions

Previous work have demonstrated remarkable antioxidant properties for HT- and HT/OLE-rich samples (i.e. C and OPE extracts, respectively) produced from OMWW by two distinct extraction systems (Foti et al., 2023). Considering these data and the abundant evidence describing olive oil phenols as capable of reducing cellular oxidative stress by stimulating mitochondrial function, herein we provide a comprehensive evaluation of the effects of C and OPE samples on continuous cell cultures.

Preliminary toxicity tests exclude any potential noxious outcome on mouse fibroblast-like cell proliferation and morphology. Moreover, pre-treatment with C and OPE samples alone, as well as with HT, protects cells from hydrogen peroxide exposure, significantly incrementing their overall survival in the long time analysis. Interestingly, the enhanced resistance to free radical damage correlates to a substantial reduction in the intracellular levels of ROS. These results are in good agreement with literature data that report HT and OLE avoided cell death and accumulation of reactive oxidant species upon pro-oxidants administration (Facchini et al., 2014; Goya, Mateos, & Bravo, 2007; Kitsati et al., 2016; Pirković et al., 2023; Rahmani et al., 2024). In our experiments, however, the presence of both phenolic compounds (i.e. OPE extract) does not provide a greater benefit to cells compared to those treated with the one carrying only hydroxytyrosol (i.e. C sample) as would be expected. It suggests that the effects of the two antioxidants are neither additive on sustaining cell viability nor on lowering ROS production upon oxidative stress induction. qPCR analysis together with western blot quantification also reveals that our OMWW extracts can stimulate mitochondrial biogenesis in NCTC cells. In this case, OPE performs better than C sample, albeit this slight difference is no longer noticeable in the quantitative analysis of mitochondrial content. Accordingly, the significative up-regulation of mitochondrial mass triggered by both phenolic specimens is roughly comparable to that observed with hydroxytyrosol alone. Our data perfectly fit with the numerous papers claiming that HT and OLE boosted the growth and division of mitochondria in different cell models (Chodari et al., 2021; Hao et al., 2010). The overexpression of the transcriptional coactivator PGC-1α that we registered also emphasizes the interplay between mitochondrial metabolism and oxidative stress. Apart from being a master regulator of mitochondrial biogenesis, PGC-1α has been reported to control oxidative phosphorylation and detoxification of radical species (Abu Shelbayeh, Arroum, Morris, & Busch, 2023; Rius-Pérez, Torres-Cuevas, Millán, Ortega, & Pérez, 2020). The antioxidant effect of our OMWW fractions could therefore be related, at least in part, to the increase in the amount of PGC-1α, which in turn, suppresses redox imbalance. Little information is available about the consequences of hydroxytyrosol and oleuropein administration on mitochondrial respiration, particularly following an oxidative stimulus. In (Hao et al., 2010) authors demonstrated that HT increased the expression and the activities of Complex I,II, III, and V as well as oxygen consumption in mouse adipocytes. Also recently, OLE proved to reduce mitochondrial impairment and enhance respiratory chain Complex I, IV, V activity upon rotenone-induced toxicity (Singh et al., 2023). The HRR experiments we conducted show that C and OPE samples, analogously to HT alone, do not ameliorate the phosphorylation efficiency of NCTC mitochondria under non-stress conditions. Rather, in these circumstances, C sample decreases oxygen consumption, negatively affecting various bioenergetics parameters such as ROUTINE and maximal ET capacity. Notwithstanding, pre-treatment with HT and, to a lesser extent with OPE sample, considerably mitigates the mitochondrial respiration impairment triggered by H_2_O_2_ addition by improving the overall oxygen consumption and, at the same time, by increasing the reserve respiratory capacity. In accordance with (Martins, Gomes-Laranjo, Amaral, Almeida, & Peixoto, 2008; Peixoto et al., 2008), the opposite effects of our OMWW fractions on mitochondrial bioenergetics could be attributable to the considerable amount of organic acids (i.e. propionic, lactic, and acetic acids) existing in the C sample in contrast to OPE, which includes only a low content (1356.50 mg/L) of propionic acid (Suppl. Fig.1). The incorporation of organic compounds into mitochondrial membranes has been indeed proposed to be the reason for the uncoupling effect of olive mill wastewater on oxidative phosphorylation. Consistently, the resulting alterations in the chemico-physical and structural properties of the inner membrane would hamper the electron passage between redox complexes and, in addition, aggravate the proton leak (Martins et al., 2008; Peixoto et al., 2008). As a matter of fact, accumulation of organic acids, in particular lactic acid, has been associated with mitochondrial dysfunction in fibroblasts from patients with idiopathic Parkinson's disease (iPD) (Juárez-Flores et al., 2020).

Taken together, data reported here demonstrate that both examined OMWW extracts overcome ROS imbalance due to oxidative stress induction and stimulate mitochondrial biogenesis thanks to their phenolic composition, though only OPE sample is able to sustain mitochondrial respiration upon free radical injury. In this regard, the selective resin extraction undoubtedly stands out as the best method for recovering phenol compounds from olive oil waste. Nevertheless, it is worth noting that organic acids also offer some advantages: they can improve the taste of food, maintain nutritional value and extend shelf life by inhibiting bacterial division and proliferation, hence functioning as **bio-preservatives (**Shi, Pu, Zhou and Zhang, 2022). In a context of sustainability, C and OPE samples could be suitable for two different market segments: the former could be used as a new food additive providing a green label for the consumer, while the latter as a food supplement with high nutraceutical value.

In conclusion, our work provides new insights into the biological activities of natural phenolic antioxidants from olive mill waste water, highlighting for the first time their strength in preventing mitochondrial respiration impairment provoked by a pro-oxidant stimulus. Moreover, it suggests different applications of OMWW extracts according to the method used for their production and opens up new perspectives to improve our phenolic-rich fractions with the aim of promoting their employment in the food and nutraceutical industries.

The following are the supplementary data related to this article.

## Authors contributions

I.R.I performed cell cultures and western blot. S.A.M.C executed high resolution respirometry measurements and prepared figs. S.C.N. performed high-resolution respirometry experiments and analyzed the results. P.F. prepared the OMWW extracts and examined their chemical composition. M.F.T. conducted the Incucyte experiments. S.B. carried out cell cultures. G.B. performed high- resolution respirometry experiments. M.F.T., A. Magrì, A. Messina, F.V.R., C.C. and V.D.P. reviewed and edited the text. S.R. and V.D.P. wrote the original draft of the manuscript. S.R. conceived and planned the experiments. All authors discussed the results and contributed to the final manuscript.

## CRediT authorship contribution statement

**Iolanda Rita Infantino:** Methodology, Investigation. **Salvatore Antonio Maria Cubisino:** Methodology, Investigation, Data curation. **Stefano Conti Nibali:** Methodology, Investigation, Data curation. **Paola Foti:** Methodology, Investigation. **Marianna Flora Tomasello:** Writing – review & editing, Validation, Investigation. **Silvia Boninelli:** Methodology, Investigation. **Giuseppe Battiato:** Methodology, Investigation. **Andrea Magrì:** Writing – review & editing, Validation, Investigation, Data curation. **Angela Messina:** Writing – review & editing. **Flora Valeria Romeo:** Writing – review & editing. **Cinzia Caggia:** Writing – review & editing, Funding acquisition. **Vito De Pinto:** Writing – review & editing, Writing – original draft, Funding acquisition. **Simona Reina:** Writing – review & editing, Writing – original draft, Validation, Supervision, Project administration, Data curation, Conceptualization.

## Declaration of competing interest

The authors declare that they have no known competing financial interests or personal relationships.

that could have appeared to influence the work reported in this article.

## Data Availability

No data was used for the research described in the article.
